# Insights into the relationship between ferroelectric and photovoltaic properties in CsGeI_3_ for solar energy conversion

**DOI:** 10.1039/d2ra06860e

**Published:** 2023-01-11

**Authors:** N. Chelil, M. Sahnoun, Z. Benhalima, R. Larbi, Sayed M. Eldin

**Affiliations:** a Laboratoire de Physique Quantique de la Matière et Modélisation Mathématique (LPQ3M), University of Mascara Algeria msahnoun@univ-mascara.dz; b Center of Research, Faculty of Engineering, Future University in Egypt New Cairo 11835 Egypt

## Abstract

Materials such as oxide and halide perovskites that simultaneously exhibit spontaneous polarization and absorption of visible light are called photoferroelectrics. They hold great promise for the development of applications in optoelectronics, information storage, and energy conversion. Devices based on ferroelectric photovoltaic materials yield an open-circuit voltage that is much higher than the band gap of the corresponding active material owing to a strong internal electric field. Their efficiency has been proposed to exceed the Shockley–Queisser limit for ideal solar cells. In this paper, we present theoretical calculations of the photovoltaic properties of the ferroelectric phase of the inorganic germanium halide perovskite (CsGeI_3_). Firstly, the electronic, optical and ferroelectric properties were calculated using the FP-LAPW method based on density functional theory, and the modern theory of polarization based on the Berry phase approach, respectively. The photovoltaic performance was evaluated using the Spectroscopic Limited Maximum Efficiency (SLME) model based on the results of first-principles calculations, in which the power conversion efficiency and the photocurrent density–voltage (*J*–*V*) characteristics were estimated. The calculated results show that the valence band maximum (VBM) of CsGeI_3_ is mainly contributed by the I-5p and Ge-4s orbitals, whereas the conduction band is predominantly derived from Ge-4p orbitals. It can be seen that CsGeI_3_ exhibits a direct bandgap semiconductor at the symmetric point of *Z* with a value of 1.53 eV, which is in good agreement with previous experimental results. The ferroelectric properties were therefore investigated. With a switching energy barrier of 19.83 meV per atom, CsGeI_3_ has a higher theoretical ferroelectric polarization strength of 15.82 μC cm^−2^. The SLME calculation also shows that CsGeI_3_ has a high photoelectric conversion efficiency of over 28%. In addition to confirming their established favorable band gap and strong absorption, we demonstrate that CsGeI_3_ exhibits a large shift current bulk photovoltaic effect of up to 40 μA V^−2^ in the visible region. Thus, this material is a potential ferroelectric photovoltaic absorbed layer with high efficiency.

## Abstract 2

In addition to its favorable band gap and strong absorption, CsGeI_3_ exhibits a large shift current bulk photovoltaic effect of up to 40 μA V^−2^ in the visible region.

## Graphical abstract

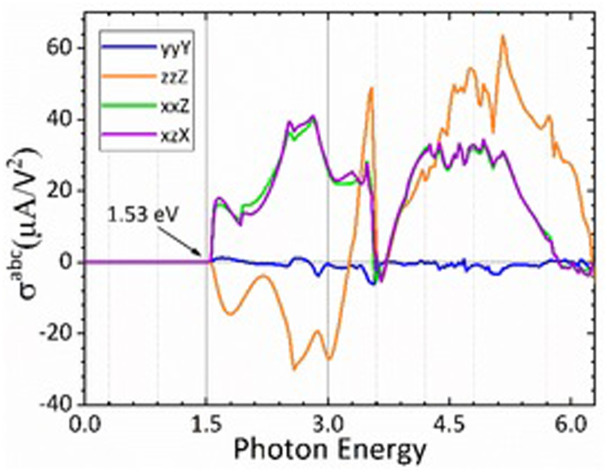

## Introduction

Solar energy technology has been continuously improved as part of renewable energy technology. A solar cell, commonly known as a photovoltaic cell, is the most widely used device that converts sunlight directly into electricity. The important accomplishments of halide perovskites in photovoltaics and other optoelectronic applications result from a great advantageous combination of optical and electronic properties and their simple fabrication processes. This distinguishes them as an entirely new class of semiconductors that can approach or exceed the performance of the typical III–V and II–IV semiconductor classes that still rule the applied optoelectronic industry.^1–7^ The emerging halide perovskites with exceptional semiconducting properties also have the potential to be ferroelectric. Recently, it was suggested that the high performance of photovoltaic cells based on the use of halide perovskites originates from the presence of ferroelectricity in these perovskites. Some theoretical works have shown that ferroelectricity in halide perovskites reduces the recombination of electron–hole pairs excited by light, and also extends the charge carrier lifetimes to several nanoseconds.^4^ Consequently, ferroelectricity is a good approach to increase photovoltaic performance beyond the Shockley–Queisser limit. The photovoltaic response in ferroelectric materials is called “the bulk photovoltaic effect” (BPVE). The photocurrent of the ferroelectric–photovoltaic device is governed by the light-absorption process, exciton dissociation efficiency, the lifetime of the photogenerated nonequilibrium charges, and charge carrier mobility. The Shift current which is the dominant photocurrent response in the bulk photovoltaic effect (BPVE) may be calculated using the first-principles method, which uses perturbation theory to estimate the transition intensity and shift vector.^8–12^ The measurements conducted on the tetragonal methylammonium lead triiodide (CH_3_NH_3_PbI_3_) confirm its excellent light-absorbing and charge-transporting properties, which make it ideal to give crucial insight into the design and working mechanism of perovskite solar cells. At low temperatures, Cahen *et al.* reported experimental findings of ferroelectric hysteresis in tetragonal CH_3_NH_3_PbI_3_ single crystals,^13^ and they express all of the necessary ferroelectricity-related features: lack of inversion symmetry, spontaneous polarization, and presence of polar domains. In addition, Huang *et al.* showed the existence of ferro-elastic domains in polycrystalline CH_3_NH_3_PbI_3_ films and single crystals.^14^ Frost *et al.* calculated the polarization magnitude of CH_3_NH_3_PbI_3_ perovskite up to 38 μC cm^−2^ (similar to that of the ferroelectric oxide perovskite KNbO_3_).^1^ The electric polarization present in the ferroelectric CH_3_NH_3_PbI_3_ has been shown to effectively separate the photoexcited carriers, leading to novel ferroelectric PV materials with potentially enhanced energy conversion efficiency. Lead toxicity which has hazardous effects on the environment and the human body has drawn considerable attention to emerging halide perovskite solar cells. To address this toxicity issue, intensive research efforts have recently been made to replace lead with non-toxic elements without compromising the photovoltaic properties. So far, various metal cations such as tin or germanium have been explored as alternatives for the development of lead-free halide perovskites. High-throughput computational methods based on density functional theory (DFT) have been proposed to search for a suitable replacement for lead in halide perovskite materials, drawing attention to their computed band gap values and their potential solar cell applications. Krishnamoorthy *et al.*^15^ investigated a series of AMX_3_ chemical compositions with A^1+^ and X^1−^ ions selected from {K, Rb, and Cs} alkali metals and {Cl, Br, and I} halogen elements, respectively, in combination with various divalent candidates *M* site cations. The inorganic germanium perovskite compound CsGeI_3_ was identified among these compounds as the energetically most stable. The first synthesis and properties of CsGeI_3_ were reported by Stoumpos *et al.*,^16^ and through the X-ray diffraction characterization, the compound has been indexed to a rhombohedral crystal structure (with a *R*3*m* space group symmetry), which shows intriguing ferroelectric properties. The theoretical power conversion efficiency (PCE) of CsGeI_3_ in photovoltaic perovskite solar cells has increased rapidly, from around 18% to more than 31%.^17–19^ Because of its suitable charge mobility and low hole-effective mass, it has been proposed as an efficient hole transport material that can enhance the efficiency. The experimental findings of CsGeI_3_ were also published by Chen *et al.*,^20^ who examined its power conversion efficiency (PCE), which reached 4.92% under AM1.5G. Similarly, Krishnamoorthy *et al.*^15^ evaluated its PCE to be 11%. Understanding the linear and nonlinear optical (NLO) responses of the ferroelectric phase of CsGeI_3_ is critical for its effective use in solar applications. Therefore, in this paper, the FP-LAPW method based on density functional theory and the modern theory of polarization based on the Berry phase approach were used to calculate the optical and ferroelectric properties of CsGeI_3_, respectively. Secondly, based on these computed findings, the photoresponse and photovoltaic properties were investigated using the Spectroscopic Limited Maximum Efficiency (SLME) model, in which the power conversion efficiency and the photocurrent density–voltage (*J*–*V*) characteristics were evaluated. We will demonstrate that the ferroelectric phase of CsGeI_3_ can exhibit suitable nonlinear optical responses in the visible region, which is a potential mechanism for the high photovoltaic efficiency of next-generation solar cells.

## Computational methodology

The calculations reported in this work have been performed using the full-potential linear-augmented plane waves plus local orbitals (FP-LAPW) method, within the generalized gradient approximation (GGA) as implemented in the Wien2k code.^21,22^ The Tran-Blaha modified Becke-Johnson (TB-mBJ) potential was used to find out the suitability for the study of electronic and optical properties of the CsGeI_3_ compound.^23–27^ LAPW sphere radii of 2.4, 2.3, and 2.4 bohr were employed for Cs, Ge, and I, respectively. The geometric structure was relaxed until the total energy change was less than 10^−5^ eV, and the forces acting on the atoms were relaxed to less than 0.001 eV Å^−1^. One of the most important properties of ferroelectric materials is spontaneous polarization. It is defined as the shift in polarization that happens when a crystal transforms from a centrosymmetric structure to a structure lacking inversion symmetry. For our study, we employed the program BerryPI^28^ to calculate polarization using the Berry phase method.^29,30^ A form of photovoltaic effect that occurs in certain semiconductors and insulators is the bulk photovoltaic effect (BPVE). Shift current is an intriguing concept for BPVE. This is a second-order process involving electron wavefunctions that have been excited twice by light. The first-principles method may be used to compute the transition intensity and shift vector based on perturbation theory in order to derive short-circuit current. Wannier90, which is based on maximally localized Wannier functions (MLWFs),^31^ was used to perform the shift current, which is closely related to the Wannier interpolation method. The MLWFs were calculated by projecting a series of Bloch energy bands into s-like and p-like orbitals on Ge and I atoms. The photovoltaic performance was evaluated using the Spectroscopic Limited Maximum Efficiency (SLME) model based on the results of first-principles calculations.^32^ It can be used for an initial screening based only on intrinsic properties. This means that greater theoretical efficiency can be obtained within this approach simply by calculating the *J*–*V* characteristic with an absorption spectrum.^33–36^

## Results and discussion

### Crystal structure and electronic band structure

Firstly, we perform the full geometry optimization to determine the crystal parameters in the rhombohedral phase of CsGeI_3_. The optimized unit-cell parameters and bond lengths are summarized in Table 1. According to the optimized geometry, we have performed the electronic structure calculations of the rhombohedral phase of CsGeI_3_ based on the FP-LAPW method^21^ and by using Tran and Blaha's^23^ modified Becke-Johnson potential functional, denoted TB-mBJ. Unlike standard GGA functionals, which are optimized to reproduce total energies, these potential delivers band gaps that are consistent with experiments for many basic semiconductors and insulators.^24–26,37–39^

**Table 1:** The calculated lattice constants and coordinates of the rhombohedral phase of CsGeI_3_ (space group *R*3*m*). Hole and electron effective masses (
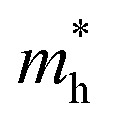
 and 
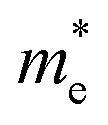
) along different directions for CsGeI_3_ (*m*_0_ is the electron rest mass). The VBM and the CBM are both located at the *Z* point

	*a* (Å)	G–I bond-length (Å)		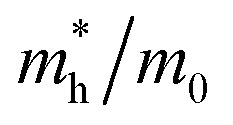		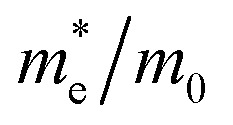	
		Short	Long	*Z*–*L*	*Z*–*Γ*	*Z*–*L*	*Z*–*Γ*
Our work	8.34	2.78	3.35	0.26	0.24	0.43	0.347
Others	8.34^a^	2.75^a^	3.26^a^	0.22^b^	0.21^b^	0.42^b^	0.21^b^

a Ref. 16.

b Ref. 17.

**Fig. 1:**
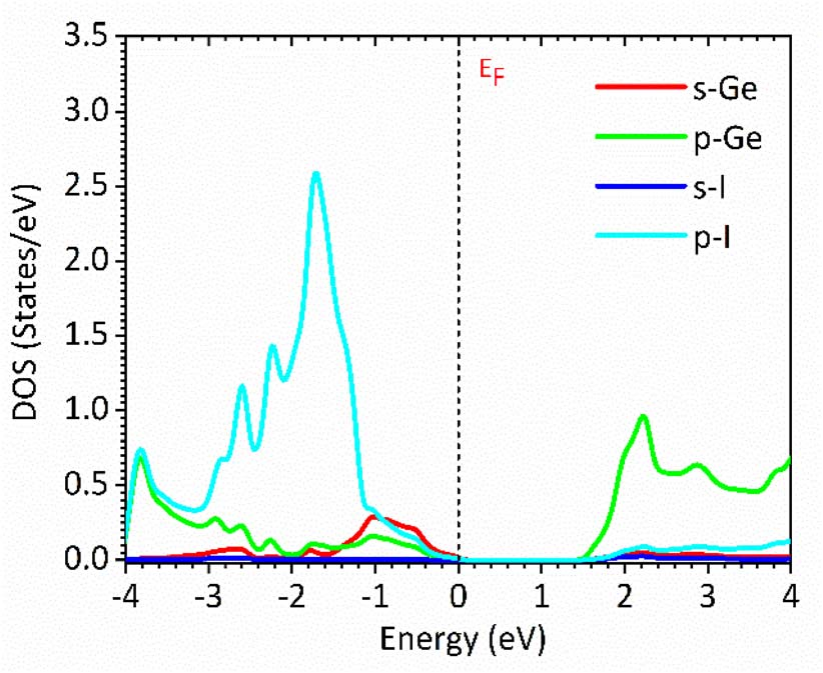
Partial density of states (DOSs) of the rhombohedral phase of CsGeI_3_ based on FP-LAPW within TB-mBJ with the Fermi levels at 0 eV.

**Fig. 2:**
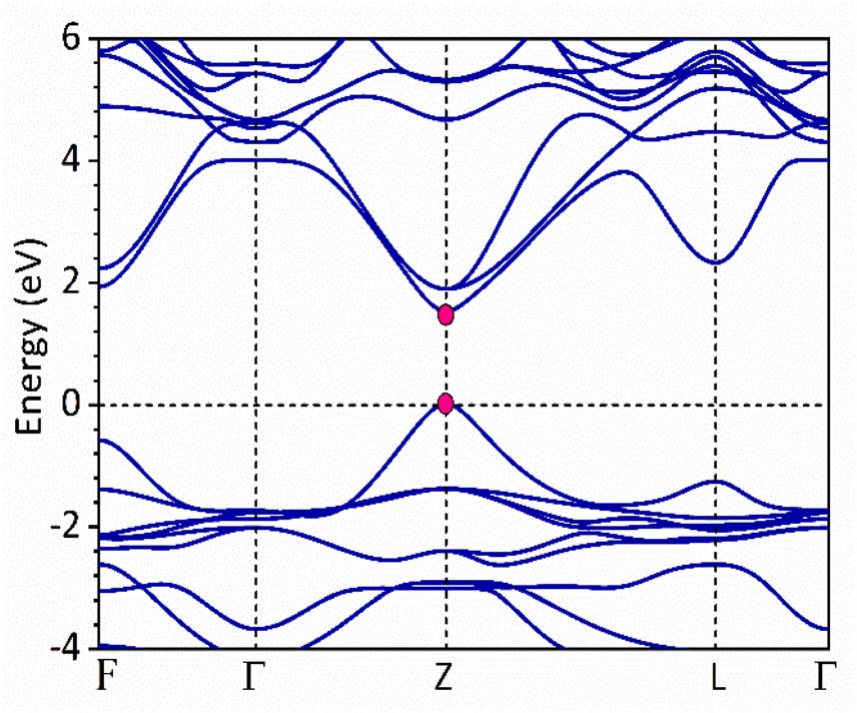
The band structure of the rhombohedral phase of CsGeI_3_ along the high symmetry directions in the Brillouin zone based on FP-LAPW within TB-mBJ.

Fig. 1 and 2 show the density of states and the energy band structure of this compound along the high symmetry points in the first Brillouin zone, respectively. From Fig. 1, the valence band maximum (VBM) of CsGeI_3_ is mainly contributed by the I-5p and Ge-4s orbitals, whereas the conduction band is predominantly derived from Ge-4p orbitals. The states of Cs atom are far from the Fermi level and do not contribute to any of the band edge states. From Fig. 2, it can be seen that CsGeI_3_ is a direct bandgap semiconductor at the symmetric point of *Z* with a value of 1.53 eV, which is in good agreement with previous experimental results.^15–17^ The optoelectronic properties significantly depend on carrier mobilities, which are closely related to the effective masses of carriers. We, therefore, calculate the effective masses of CsGeI_3_ by fitting the energy dispersions of VBM and CBM to parabolic functions along different *k* directions in the vicinity of the direct bandgap edges according to the following equation.^40^1
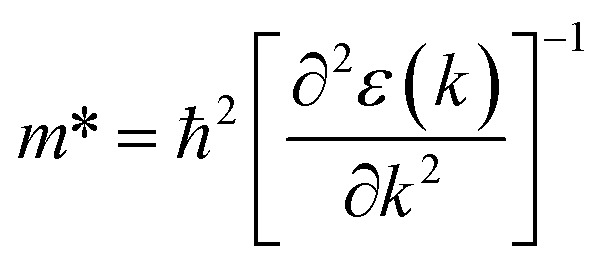


The *k* is the wave vector along different directions. The symbol *ħ* and *ε*(*k*) represent the Planck constant and eigenvalues of the energy band, respectively.

The calculated effective masses for holes in *x*, *z* directions are about 0.26, which shows weak anisotropy in hole effective masses. As for the electron effective masses, the anisotropy is obvious. The electron effective masses along [100] and [001] directions are 0.43 and 0.347, respectively. The calculated effective masses for both electrons and holes (∼0.25–0.4), indicate the good transport properties of the rhombohedral phase of CsGeI_3_.

### Optical properties

The investigation of the optical properties of CsGeI_3_ provides useful information regarding its application in optoelectronic devices. The optical response of the medium is determined by its dielectric function *ε*(*ω*) = *ε*_1_(*ω*) + i*ε*_2_(*ω*) at all photon energies. Since the imaginary part of the dielectric function *ε*_2_(*ω*), which is an important quantity for the optical transitions between energy bands, is directly related to the electronic band structure of a material, it can therefore be calculated using the information from wave functions and energies approximated from solutions of the Kohn–Sham equation:^41^2
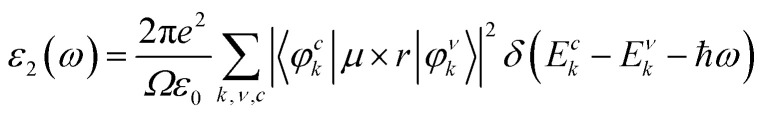
where *ε*_0_ is the vacuum dielectric constant, *Ω* is the volume, *ν* and *c* represents the valence and conduction bands, respectively, *ħω* is the energy of the incident photon, *u* is the vector defining the polarization of the incident electric field, *u* × *r* is the momentum operator, and *φ*^*c*^_*k*_ and *φ*^*ν*^_*k*_ are the wave functions of the conduction band and the valence band at the *k* point, respectively. In this equation, the integration is done in the first Brillouin zone. The Kramers–Kronig relations can be used to establish relationship between the dispersions of the real and imaginary parts of the dielectric function:3
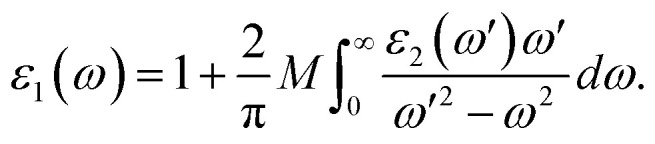


The elements of the dipole momentum *M*_*cv*_(*k*) = *φ*^*c*^_*k*_|*δ*·∇|*φ*^*ν*^_*k*_ are the elements of the direct transition matrix between the valence band *φ*^*ν*^_*k*_ and the conduction band *φ*^*c*^_*k*_, in which *δ* is the potential vector describing the electric field. The absorption spectrum indicates the light-gathering ability of a material. The absorption coefficient is calculated by the following relation;^42^4
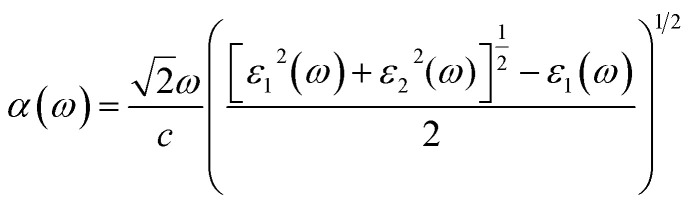
or, in term of energy (*E*)5
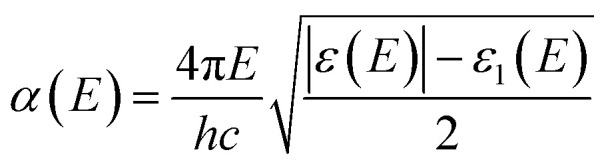
with *h* is the Planck constant and *c* is the speed of light.

**Fig. 3:**
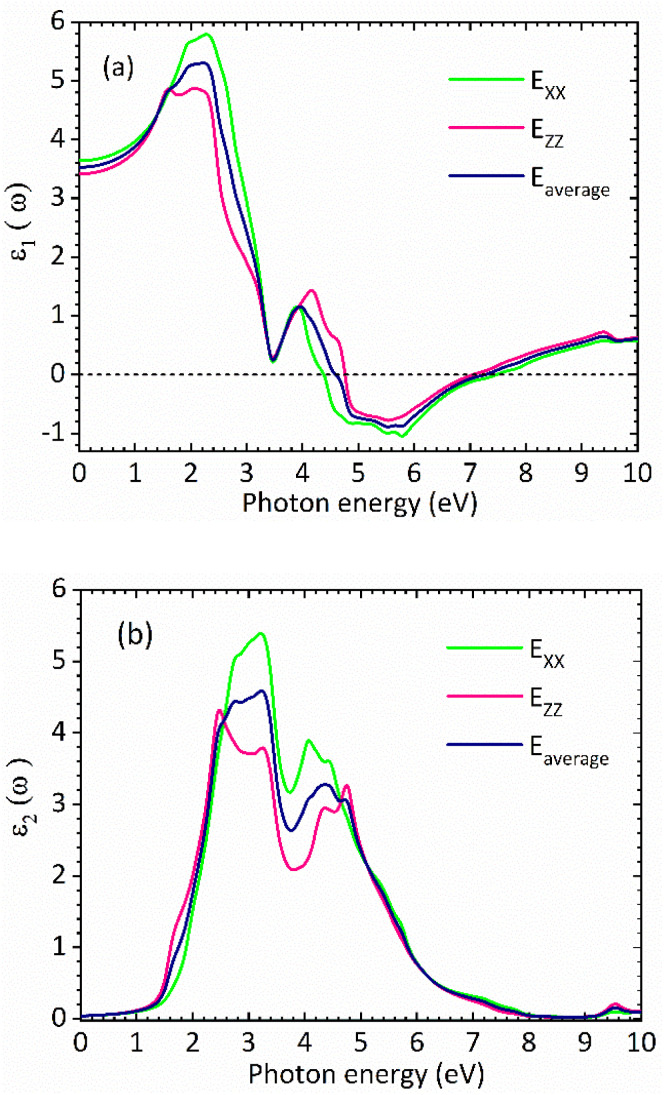
Calculated (a) real part and (b) imaginary part of the dielectric function of the rhombohedral phase of CsGeI_3_.

Fig. 3(a) and (b) show the real and the imaginary parts of the dielectric function spectra for rhombohedral phase of CsGeI_3_, respectively. The maximum value for the real part of dielectric function *ε*_1_ is achieved at around 2.27 eV. The higher value of *ε*_1_ shows a greater ability for the polarization of low incident photon energy. Moving towards higher incident photon energy, an inflection point is observed around 5.78 eV, where *ε*_1_ takes on negative values. Above 5.78 eV, its intensity increases from negative to positive values. The imaginary part of the dielectric function, *ε*_2_, is shown in Fig. 3(b). This component is related to the transitions between the valence and conduction bands, and therefore to the electronic structure of CsGeI_3_ and the absorption of the incident photons.

**Fig. 4:**
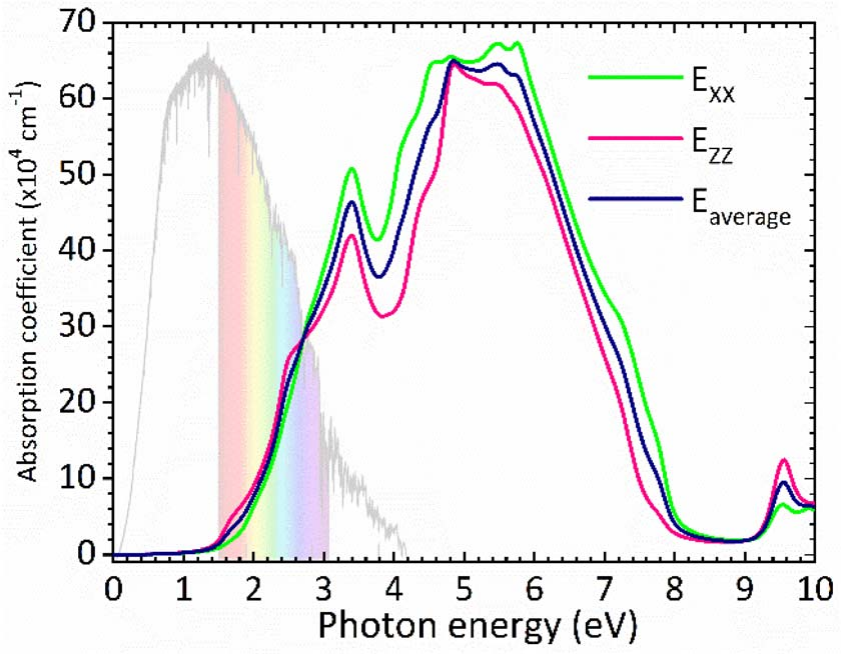
Calculated absorption spectrum for rhombohedral phase of CsGeI_3_.

The optical band gap of CsGeI_3_ can then be estimated from *ε*_2_ to a value of 1.53 eV comparable to the electronic band gap energy obtained from the electronic structure. Above the optical band gap energy, a sharp rise in the intensity *ε*_2_ is observed, with two successive strongest peaks appearing at 3.22 eV and 4.07 eV, which mainly originated from the electron transitions between the Ge 4s valence band to the Ge 4p conduction band, as indicated by the density of states of CsGeI_3_ (Fig. 1). Fig. 4 shows the calculated absorption spectrum for rhombohedral phase of CsGeI_3_. The optical absorption records a strong onset of direct transitions at ∼2.9 eV, which can demonstrate that the rhombohedral phase of CsGeI_3_ shows a great sunlight absorption in the visible region.

### Spectroscopic limited maximum efficiency (SLME)

An effective way to evaluate the maximum efficiency of a photovoltaic cell using a single p–n junction is by using the Shockley–Queisser limit.^43^ The Shockley–Queisser limit is calculated by examining the amount of electrical energy that is extracted per photon of incoming sunlight. It offers a relationship between the bandgap of a material and its maximum efficiency. Yu and Zunger^32^ have recently extended the work of Shockley and Queisser by employing the Spectroscopic Limited Maximum Efficiency (SLME) and including the absorption spectrum and the absorber layer thickness in the efficiency assessment. The SLME has been successfully applied to a wide range of solar absorber materials, including perovskites,^44–47^ chalcogenides,^48,49^ and other materials.^50–55^ Theoretically, the maximum solar cell efficiency is defined as^56,57^6
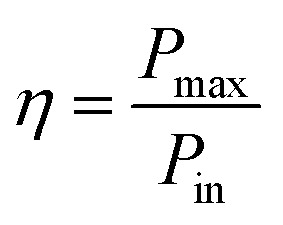
where *P*_max_ is the maximum power density and *P*_in_ is the total incident power density from the solar spectrum. The maximum power density is derived using the *J*–*V* characteristic of the solar cell:7
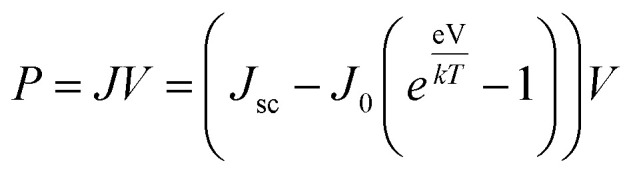
with *J* the total current density, *V* the potential over the absorber layer, *k* Boltzmann's constant, *T* the temperature of the device and *e* the elementary charge. The short circuit current density *J*_sc_ and the reverse saturation current density *J*_0_ are calculated from the absorbance *A*(*E*) of the material, as well as the AM1.5G solar spectrum *I*_sun_(*E*) and the black-body spectrum *I*_*bb*_(*E*,*T*):8
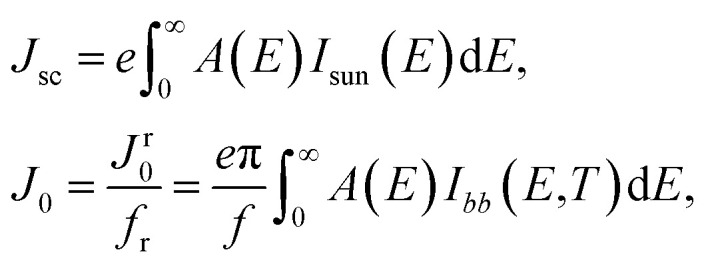
where *J*^r^_0_ is the radiative recombination current density. The absorbance *A*(*E*) for an absorber layer of thickness *L* with a reflecting back surface is defined as:^21^

9 *A*(*E*) = 1 − e^−2*α*(*E*)*L*^

The absorption coefficient *α*(*E*) is given by eqn (5). The absorbance *A*(*E*) in eqn (9) increases with the thickness *L* because there is an increasing chance to be absorbed when light travels further inside the material. The fraction of radiative recombination *f*_r_ is modeled using a Boltzmann factor:10
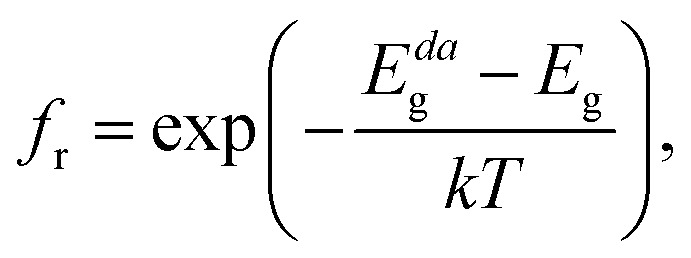
where *E*_g_ and *E*^*da*^_g_ are respectively the fundamental and direct allowed band gaps.

The open-circuit voltage *V*_oc_ which corresponds to the maximum voltage available from a solar cell, and this occurs under zero current density condition, is determined as follows,11
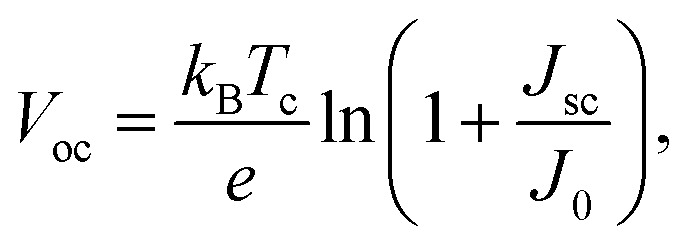


The fill factor (FF) is essentially a measure of the quality of the solar cell. It is calculated by comparing the maximum power that would be delivered from or to a device at both open circuit voltage and short circuit current combined.12
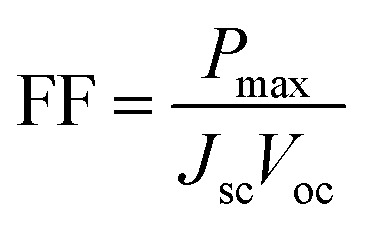


After we calculate the band gap and dielectric function for the rhombohedral phase of CsGeI_3_ compound, we have all the required information to calculate its SLME.

Because we find a direct allowed fundamental band gap for CsGeI_3_, we only have to consider cases where the non-radiative recombination is negligible (*f*_r_ = 1, see eqn (10)).

**Fig. 5:**
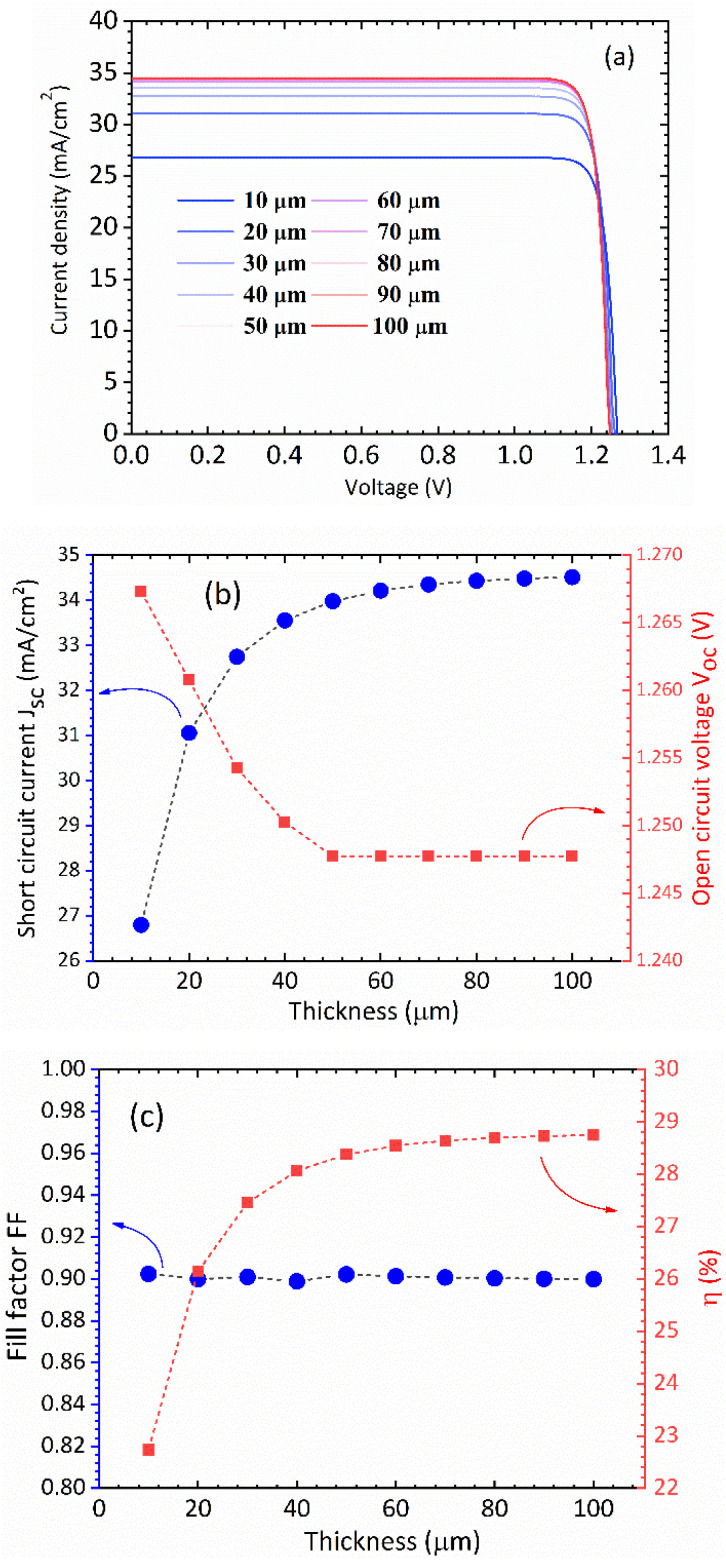
(a) Calculated *J*–*V* characteristic as a function of absorber layer thickness of the rhombohedral phase of CsGeI_3_ at *T* = 300 K. Effect of absorber layer thickness on (b) *J*_sc_ and *V*_oc_, and (c) fill factor and power conversion efficiency.

In Fig. 5(a) we show the calculated *J*–*V* characteristic of the rhombohedral phase of CsGeI_3_. These results clearly show an increasing *J*_sc_ with absorber thickness and decreasing *V*_oc_ (Fig. 5(b)). The total current density *J* remains close to *J*_sc_ up to a certain voltage *V*_m_ that maximizes the power density. The maximum solar cell efficiency *η* and the fill factor are plotted as a function of the film thickness in Fig. 5(c). We can see that for CsGeI_3_, the fill factor remains almost constant around the value of 0.89. The efficiency rises quickly for an increasing thickness. For an infinitely thick absorption layer the curve becomes a step function. This curve indicates that at a thickness of 70 μm or greater, the cell efficiency maintains its highest value *η*_max_ of around 28.7% due to maximum absorption of light as evidenced from constant *J*_sc_ (Fig. 5(b)). This highlights the potential of the rhombohedral phase of CsGeI_3_ as absorber layers in thin-film solar cells.

We present the calculated efficiency values in Table 2, in order to compare our results with those of Yu and Zunger, all efficiencies were evaluated using thickness *L* = 50 μm. These results clearly show that the rhombohedral phase of CsGeI_3_ has a favourable optical gap for solar light absorption in which the absorber thickness of 600–700 nm is suitable. Therefore, there is no benefit in using thicker (>700 nm) films.

**Table 2:** Calculated solar cell parameters for CsGeI_3_, compared with other compounds from literature, all efficiencies were evaluated using thickness *L* = 50 μm

Cells	PCE (%)	*V*_oc_ (V)	*J*_sc_ (mA cm^−2^)	FF	*E*_g_ (eV)
CsGeI_3_	28.37	1.24	33.98	0.899	1.53
Si^a^	26.70	0.738	42.65	0.849	1.12
GaAs^a^	29.10	1.127	29.78	0.867	1.42
PSCs^a^	25.20	1.181	25.14	0.848	1.56
AgGaSe_2_^b^	27.00	—	—	—	1.41
CuGaSe_2_^b^	27.80	—	—	—	1.19
CuGaTe_2_^b^	28.90	—	—	—	1.06
AgGaTe_2_^b^	28.90	—	—	—	0.95
CuInS_2_^b^	27.90	—	—	—	0.94
CuInSe_2_^b^	20.70	—	—	—	0.58

a Ref. 56.

b Ref. 57.

### Ferroelectric properties

**Fig. 6:**
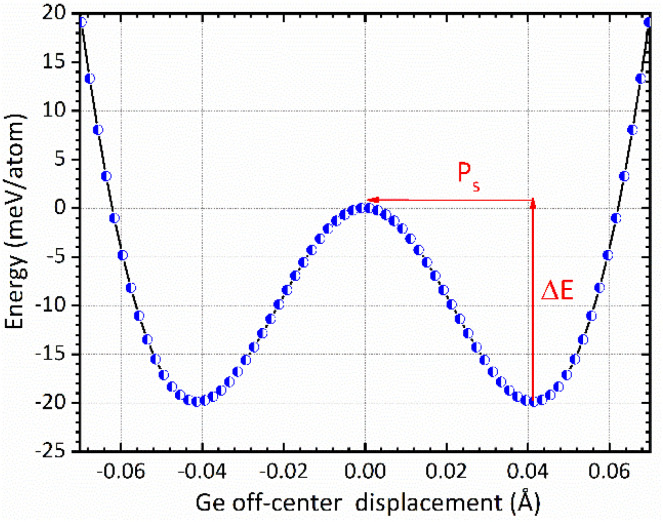
The change of total energy as a function of the relative location of the Ge^2+^ ion within the halides (*I*) octahedral cage in the rhombohedral phase of CsGeI_3_. The centrosymmetric phase is taken as energy reference.

The mechanism of ferroelectricity in CsGeI_3_ has not yet been described in detail. CsGeI_3_ is one of the all-inorganic metal halide perovskite materials in which the presence of Ge lone pairs could lead to lattice instabilities that trigger the breaking of inversion symmetry and allow the appearance of permanent electric dipoles. Hence, it shows a rhombohedral structure that is slightly deformed from the cubic perovskite structure along its diagonal direction. Therefore, symmetry breaking in the CsGeI_3_ ferroelectric phase (polar phase) is driven by zone-center lattice instability of the centrosymmetric (paraelectric, non-polar) phase, which shows collective displacement of Ge ions away from the GeI_6_ octahedral centers. The amplitude of the displacement is an important factor in determining the ferroelectric properties. Fig. 6 presents the symmetric double-well depth curve indicating the change of total energy as a function of the relative location of the Ge^2+^ ion within the halides (*I*) octahedral cage. This curve is a quantitative indicator of the energetic stability of the polar (ferroelectric) phase over the non-polar (paraelectric) phase with an energy barrier (Δ*E*) of 19.83 meV per atom. Similar representation has been reported by Chen *et al.*^58^ and Zhang *et al.*^59^ for the polar tetragonal BaTiO_3_. The low energy barrier of CsGeI_3_ may support the fast ferroelectric switching in experiments. The faster switching side states can be manipulated by applying an electric field in a specific direction. Further investigations are needed to confirm the fast ferroelectric switching of CsGeI_3_ in a way that makes it a viable candidate for low-power computing and electronics. In the absence of external applied electric field, ferroelectric materials exhibit spontaneous polarization that can be reversed by such a field. In the context of the modern theory of polarization, Berry phase calculations have been used to determine the magnitude of spontaneous polarization (Ps).^60,61^ The theoretical value of Ps in CsGeI_3_ is predicted to be around 15.82 μC cm^−2^, which is obtained from the value of polarization corresponding to the minimum of the double well potential. Our calculation result is smaller than the experimental measurement 20 μC cm^−2^.^62^ In general, the anomaly in measured polarization values can be attributed to the quality of sample, sample orientation during measurement and defect chemistry. In addition, the difference between the two results may possibly be explained by the fact that our *ab initio* calculations were performed at 0 K whereas the experimental data were obtained at room temperature.

### Nonlinear photocurrent (bulk photovoltaic effect)

Recent studies have shown that ferroelectric materials exhibit promising features for photovoltaic devices. However, the primary mechanism proposed to explain experimental observations in the literature, is the bulk photovoltaic effect (BPVE), which is associated to the ferroelectricity of material that lack inversion symmetry, and it appears to be independent of any internal fields within the material. In addition, the voltages that the material can sustain exceed conventional limits (*i.e.*, those imposed by the material's bandgap).^63–65^ In this section, we discuss the bulk photovoltaic effect (BPVE) and put more emphasis on the so-called shift current mechanism, which offers some advantages over traditional p–n junction-based solar cells. Von Baltz and Kraut^66^ presented the shift current theory as an explanation for the BPVE in BaTiO_3_, and it was later developed within the framework of Green's functions^67^ and nonlinear optics.^68^ Young and Rappe reformulated the shift current theory and have derived an adaptive formula for efficient first-principles calculation, and they offered the first comparison of experimental BPVE data with shift current theory.^69^ A more useful formulation for shift current can be found using response functions. If we define *E*^*b*^(*ω*) as an electric field with frequency *ω* this is linearly polarized in the *b* direction, the shift current takes the form13
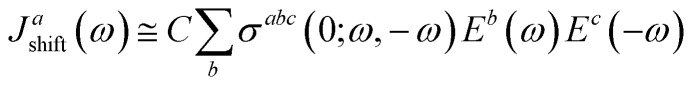
where *a*,*b*,*c* are Cartesian indices, and *σ*^*abc*^ is a third-rank tensor giving current density *J* as a response to monochromatic electromagnetic field *E*, which in a *D*-dimension is given by14
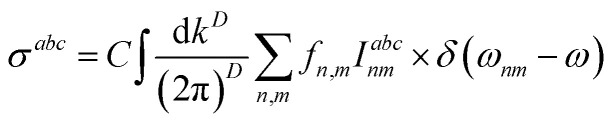
where 
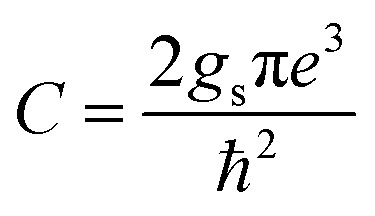
 and *g*_s_ = 2 accounts for spin degeneracy. *n*,*m* are the band indices, *f*_*nm*_ = *f*_*n*_ − *f*_*m*_ is the Fermi–Dirac occupations number of bands *n* and *m*, and *ħω*_*nm*_ = *E*_*m*_ − *E*_*n*_ is the band energy difference. The integral is over the first Brillouin zone (BZ).

15 *I*^*abc*^_*nm*_ = *r*^*b*^_*mn*_*r*^*c*^_*nm*;*a*_*R*^*a*^_*mn*_

The *I*^*abc*^_*nm*_ expression is composed of the effective position matrix elements *r*^*b*^_*mn*_ and the so-called “shift vector” *R*^*a*^_*mn*_:16
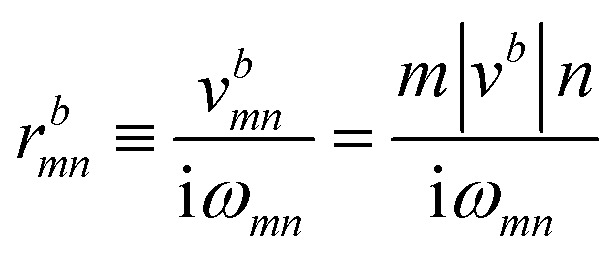
17
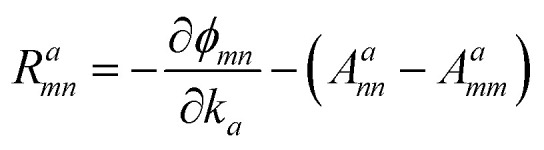
Here, *v*^*b*^_*mn*_ are velocity matrix elements, *A*^*a*^_*mm*_ are Berry connections for the band *m*, and *ϕ*_*mn*_ is the phase of the momentum matrix element between bands *m* and *n*. *r*^*b*^_*nm*_ are the inter-band matrix elements of the position operator called inter-band Berry connections,^70^ which are defined as:18
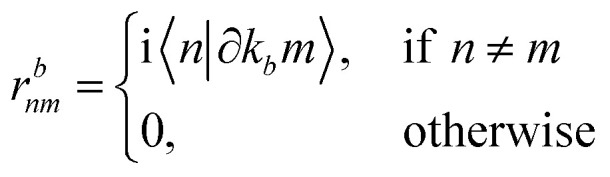
and *r*^*c*^_*nm*;*a*_ = *∂*_*k*_*a*__*r*^*c*^_*nm*_ − i(*ξ*^*a*^_*nn*_ − *ξ*^*a*^_*mm*_)*r*^*c*^_*nm*_ are generalized derivatives of the berry connections. In the last expression *ξ*^*a*^_*nn*_ = i〈*n*∣∂_*k*_*a*__*n*〉 is the diagonal berry connection for the band *n*. These berry connections are closely related to Berry phases. The calculations for CsGeI_3_ were performed using its optimized geometry in the ferroelectric phase, which belongs to a polar space group of *R*3*m* with point group 3*m* that only allows eight nonzero tensor elements for its BPVE responses, which can be obtained from first principles calculations using the shift current theory. The wave functions and eigenvalues were generated using the plane-wave density functional theory (DFT) package FP-LAPW in the TB-mBJ approximation of the exchange correlation functional. Due to the symmetry of CsGeI_3_, only four independents *σ*^*xzX*^ = *σ*^*yzY*^, *σ*^*xxY*^ = −*σ*^*yyY*^ = *σ*^*xyX*^, *σ*^*xxZ*^ = *σ*^*yyZ*^ and, *σ*^*zzZ*^ tensorial components of *σ*^*abc*^(0;*ω*;−*ω*) are nonzero. Where the uppercase letter represents the direction of shift current response and the first two lower case letters represent light polarization. For a monochromatic light linearly polarized along a particular direction, we calculate longitudinal (*σ*^*zzZ*^, *σ*^*yyY*^) and transversal (*σ*^*xzX*^, *σ*^*xxZ*^) tensorial components of *σ*^*abc*^(0;*ω*;−*ω*) of the shift current responses. The longitudinal component *σ*^*zzZ*^ (*σ*^*yyY*^) represents the shift current response along the *Z*(*Y*) axis, *i.e.*, along the direction of out-of-plane (in-plane) polarization due to *zz* (*yy*) polarized light, respectively. Whereas the transversal component *σ*^*xzX*^ (*σ*^*xxZ*^) represents the shift current response along the *X*(*Z*) axis due to *xz* (*xx*) polarized light, respectively.

**Fig. 7:**
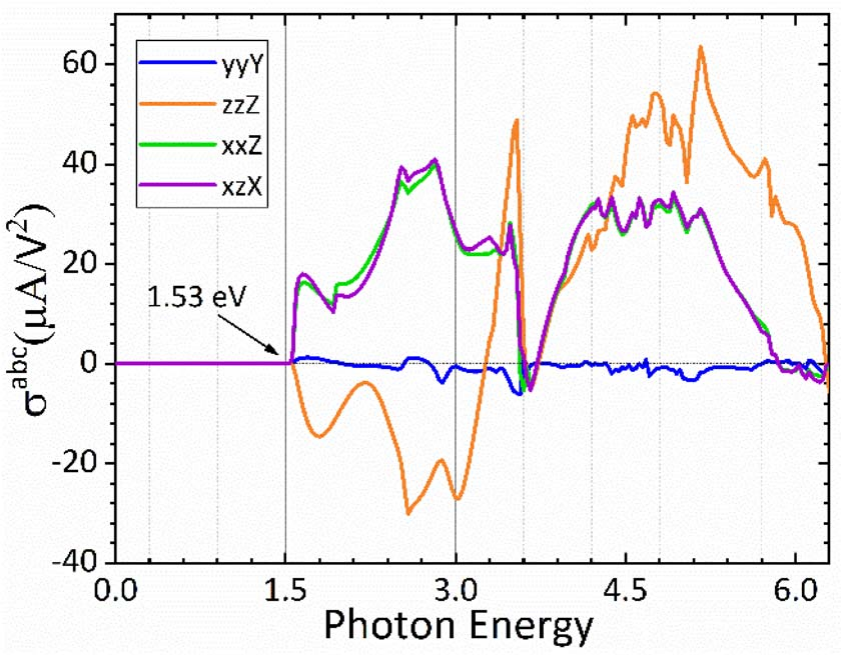
Calculated shift current tensor elements for the rhombohedral phase of CsGeI_3_.

In Fig. 7 we plot both the longitudinal and transversal shift current responses as a function of photon energy for the CsGeI_3_ compound. A scissor correction was applied to the calculated spectra in Fig. 7 to eliminate the underestimation of the gap and to facilitate its comparison with the electronic band structure. The spectral quantities were rigidly shifted by 0.362 eV. Accordingly, all shift current responses are zero below the bandgap, the dominating current responses are *σ*^*zzZ*^ and *σ*^*yyZ*^, and they all attain a maximum absolute value of about 40 μA V^−2^ above the band edge within the photon energy of 2.9 eV in the visible spectrum, which is highly desired for efficient solar energy conversion. Interestingly, the reported shift current in CsGeI_3_ is comparable to the most studied ferroelectric perovskite oxides such as BaTiO_3_ (30 μA V^−2^) and PbTiO_3_ (50 μA V^−2^).^69^ On the basis of the shift current result, we can control the direction and the magnitude of the shift current in CsGeI_3_ by tuning the applied light.

## Conclusions

First principles-based density functional theory (DFT) calculations reveal that CsGeI_3_ in its rhombohedral structure (ferroelectric phase) exhibits excellent optoelectronic properties. Specifically, the calculated results indicate that the valence band maximum (VBM) of CsGeI_3_ is mainly contributed by the I-5p and Ge-4s orbitals, whereas the conduction band is predominantly derived from Ge-4p orbitals. CsGeI_3_ exhibits a direct bandgap semiconductor with a value of 1.53 eV. The ferroelectric properties were therefore investigated. CsGeI_3_ has a higher theoretical ferroelectric polarization strength of 15.82 μC cm^−2^ related to its switching energy barrier of 19.83 meV per atom. The performance of CsGeI_3_ in photoconversion devices is analysed. Using the spectroscopic limited maximum efficiency (SLME) model, the best efficiencies of over 28% is achieved for the rhombohedral structure of CsGeI_3_, which demonstrates that lead-free germanium iodide perovskite is very good candidate for highly efficient solar energy conversion. We also apply the Wannier interpolation method to evaluate photocurrent generation in CsGeI_3_ through the bulk photovoltaic effect (BPVE). Strong absorption of visible light at ∼2.9 eV and a large shift current bulk photovoltaic effect of ∼40 μA V^−2^ is obtained in the visible region. Our findings highlight the potential of the ferroelectric structure of germanium iodide perovskite for developing third-generation solar cells with high efficiency beyond the fundamental S–Q limit.

## Author contributions

The authors confirm contribution to the paper as follows: conceptualization: N. C. and R. L.; data curation: N. C. and Z. B.; investigation: M. S. and N. C.; visualisation: Z. B.; writing – original draft: M. S., S. M. E. and N. C. All authors reviewed the results and approved the final version of the manuscript.

## Conflicts of interest

There are no conflicts to declare.

## Supplementary Material
